# A New Elementary Method for Determining the Tip Radius and Young’s Modulus in AFM Spherical Indentations

**DOI:** 10.3390/mi14091716

**Published:** 2023-08-31

**Authors:** Stylianos Vasileios Kontomaris, Andreas Stylianou, Georgios Chliveros, Anna Malamou

**Affiliations:** 1 Faculty of Engineering and Architecture, Metropolitan College, 15125 Athens, Greece; gchliveros@mitropolitiko.edu.gr; 2 BioNanoTec Ltd., Nicosia 2043, Cyprus; 3 School of Sciences, European University Cyprus, Nicosia 2404, Cyprus; stylianou.c.andreas.1@ucy.ac.cy; 4 Independent Power Transmission Operator S.A. (IPTO), 10443 Athens, Greece; a.malamou@admie.gr

**Keywords:** calibration of spherical indenters, mechanical properties, biological materials, data processing, AFM grating, intelligent AFM systems

## Abstract

Atomic force microscopy (AFM) is a powerful tool for characterizing biological materials at the nanoscale utilizing the AFM nanoindentation method. When testing biological materials, spherical indenters are typically employed to reduce the possibility of damaging the sample. The accuracy of determining Young’s modulus depends, among other factors, on the calibration of the indenter, i.e., the determination of the tip radius. This paper demonstrates that the tip radius can be approximately calculated using a single force–indentation curve on an unknown, soft sample without performing any additional experimental calibration process. The proposed method is based on plotting a tangent line on the force indentation curve at the maximum indentation depth. Subsequently, using equations that relate the applied force, maximum indentation depth, and the tip radius, the calculation of the tip radius becomes trivial. It is significant to note that the method requires only a single force–indentation curve and does not necessitate knowledge of the sample’s Young’s modulus. Consequently, the determination of both the sample’s Young’s modulus and the tip radius can be performed simultaneously. Thus, the experimental effort is significantly reduced. The method was tested on 80 force–indentation curves obtained on an agarose gel, and the results were accurate.

## 1. Introduction

Atomic force microscopy (AFM) is a powerful tool that enables imaging and mechanical characterization of soft materials at the nanoscale [1,2]. Using the AFM nanoindentation method, Young’s modulus maps on biological materials can be created and used for the diagnosis of various diseases [2,3]. In particular, groundbreaking research has shown that utilizing the AFM nanoindentation method can lead to the discrimination of cells as normal or cancerous [4]; to the characterization of human tissues as normal, benign or malignant [5,6]; to the early diagnosis of osteoarthritis [7]; to the mechanical characterization of proteins [8,9] and viruses [10]; and so on. The significant advantage of this approach lies in its user-independent disease diagnosis, which can be executed through mathematical criteria and automated computational processes. Nonetheless, several challenges remain to be addressed prior to the complete use of AFM technology in clinical activities [11]. The principal goal regarding the AFM research on biological materials is to develop a reliable system used to characterize biological materials such as cells and tissues at the nanoscale and used for medical purposes. Towards this direction it is essential to develop intelligent systems in order to reduce the complexity and the experimental effort. An AFM nanoindentation experiment, requires the calibration of the AFM tip used for the experiments (i.e., the determination of the indenter’s dimensions). In many cases, a spherical indenter is preferred, since it reduces the possibility of damaging the soft biological material. Thus, it is essential to determine the indenter’s radius prior each experiment. When testing soft biological materials at the nanoscale using spherical indenters (and assuming that the indenter is orders of magnitude stiffer than the sample), the classical Hertz equation is commonly employed for data processing [12]:

(1) $$F=\frac{4}{3}\frac{E}{\left(1-v^{2}\right)}R^{1/2}h^{3/2}$$

In Equation (1), F is the applied force on the sample, h is the indentation depth, R is the indenter’s radius, and E,v are the Young’s modulus and the Poisson’s ratio of the material, respectively. However, Equation (1) is only valid for small indentation depths compared to the tip’s radius (h≪R) [12]. The accurate equation that relates the applied force to the indentation depth was firstly derived by Sneddon and is presented below [13]:

(2) $$F=\frac{E}{2\left(1-v^{2}\right)}\left[\left(r_{c}^{2}+R^{2}\right)ln\left(\frac{R+r_{c}}{R-r_{c}}\right)-2r_{c}R\right]$$

In Equation (2), $r_{c}$ is the radius at contact depth ($h_{c}$) [14]. In addition, the indentation depth is related to the contact radius with the following equation [13]:

(3) $$ln\left(\frac{R+r_{c}}{R-r_{c}}\right)=\frac{2h}{r_{c}}$$

Equations (2) and (3) do not provide a direct relation between the applied force and the indentation depth. Thus, a new equation was recently derived [15]. The idea was to write Equation (3) as follows:

(4) $$\frac{h}{R}=\frac{1}{2}\frac{r_{c}}{R}ln\left(\frac{1+\frac{r_{c}}{R}}{1-\frac{r_{c}}{R}}\right)$$

Subsequently, the $\frac{r_{c}}{R}=f\left(\frac{h}{R}\right)$ function was fitted to a simple equation of the form [15]:

(5) $$\frac{r_{c}}{R}=c_{1}{\left(\frac{h}{R}\right)}^{1/2}+c_{2}\left(\frac{h}{R}\right)+c_{3}{\left(\frac{h}{R}\right)}^{2}+c_{4}{\left(\frac{h}{R}\right)}^{3}+\cdots +c_{N}{\left(\frac{h}{R}\right)}^{N-1}$$

In Equation (5), $c_{1},\;c_{2},\;\ldots ,\;c_{N}$ are constants that depend on the $h_{max}/R$ ratio and can be found in [15]. Equation (5) was substituted to the general differential equation that is valid for every axisymmetric indenter [16]:

(6) $$\frac{dF}{dh}=\frac{2E}{1-v^{2}}r_{c}$$

Subsequently, the solution of differential Equation (6) results in

(7) $$F=\frac{2ER}{1-v^{2}}hQ$$

In Equation (7), Q is defined as follows [15]:

(8) $$Q=\frac{2}{3}c_{1}R^{-1/2}h^{1/2}+{\frac{1}{2}c}_{2}R^{-1}h+\frac{1}{3}c_{3}R^{-2}h^{2}+\frac{1}{4}c_{4}R^{-3}h^{3}+\cdots +\frac{1}{N}c_{N}R^{1-N}h^{N-1}$$

**Figure 1:**
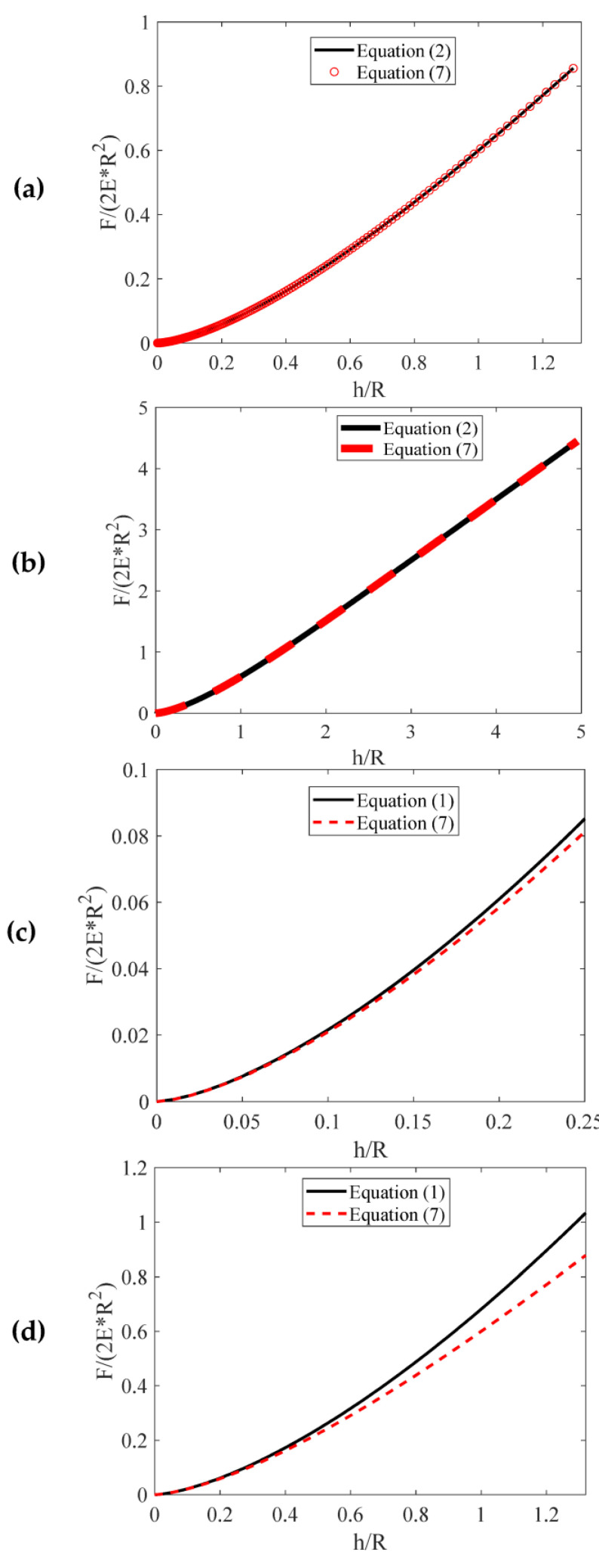
The $\frac{F}{{2E}^{*}R^{2}}=f\left(\frac{h}{R}\right)$ functions using Equations (2) and (7) for the domain (**a**) $0\leq \frac{h}{R}\leq 1.32$ and (**b**) $0\leq \frac{h}{R}\leq 4.9512$. The $\frac{F}{{2E}^{*}R^{2}}=f\left(\frac{h}{R}\right)$ functions using Equations (1) and (7) for the domain (**c**) $0\leq \frac{h}{R}\leq 0.25$ and (**d**) $0\leq \frac{h}{R}\leq 1.32$.

It is important to note that Equations (2) and (7) yield identical results, as shown in Figure 1a,b. The functions $\frac{F}{{2E}^{*}R^{2}}=f\left(\frac{h}{R}\right)$ obtained from Equations (2) and (7) are presented for comparison (where, $E^{*}=\frac{E}{1-v^{2}}$ is the sample’s reduced modulus). For 0≤h/R≤1.32, N=3 and $c_{1}=1.022$, $c_{2}=-0.1133$, $c_{3}=-0.0742$ (Figure 1a) [15]. For 0≤h/R≤4.9512, N=6 and $c_{1}=1.0100000,c_{2}=-0.0730300$, $c_{3}=-0.1357000$, $c_{4}=0.0359800$, $c_{5}=-0.0040240$, $c_{6}=0.0001653$ (Figure 1b) [15]. It is important to further note that the latter case (N=6) is applicable in any scenario [15]. However, it is too complicated. Therefore, in conventional experiments, the values N=3 and $c_{1}=1.022$, $c_{2}=-0.1133$, $c_{3}=-0.0742$ are not only precise but also greatly reduce complexity.

In addition, the $\frac{F}{{2E}^{*}R^{2}}=f\left(\frac{h}{R}\right)$ functions when using Equations (1) and (7) are shown comparatively in Figure 1c for the domain $0\leq \frac{h}{R}\leq 0.25$ and in Figure 1d for the domain $0\leq \frac{h}{R}\leq 1.32$. If Equation (1) is used instead of Equation (7) for $h_{max}/R=1$, the error in the Young’s modulus calculation will be approximately 10%. Therefore, when conducting AFM nanoindentation tests on soft materials with spherical indenters, the most suitable equation for fitting the force–indentation data is Equation (7). However, it will be demonstrated that the preference for Equation (7) over Equation (1) is not solely based on avoiding errors in Young’s modulus calculation. As already mentioned, the determination of the Young’s modulus of the tested material necessitates knowledge of the tip radius. The tip radius is usually determined using scanning electron microscopy (SEM) imaging [17] or AFM gratings [18]. This represents an extra experimental stage in AFM indentation experiments, considerably extending the time needed to determine the mechanical properties of the material. This is due to the necessity of using a new indenter for each experiment to prevent contamination or changes in the tip’s shape or dimensions (hence, prior to any experiment, a new calibration process is essential). In addition, even one additional experimental step increases the possibilities of contaminating the AFM tip.

This paper will demonstrate that by employing a single force–indentation curve, it is possible to determine both the tip radius and the Young’s modulus. Hence, the need for additional experimental processes for tip calibration can be readily circumvented. Therefore, the significant benefit of fitting the data to Equation (7), in addition to the offered accuracy in comparison to Equation (1), is the potential to conduct an AFM tip calibration without requiring any supplementary experimental procedures.

## 2. Materials and Methods

### 2.1. A New Model for Processing Force—Indentation Curves

When employing spherical indenters, the data adhere to Equations (7) and (8). However, in the majority of cases, the maximum indentation depth is $h_{max}\leq R$. It has been previously demonstrated that for $\frac{h_{max}}{R}\leq 1.32$ [15]:

(9) $$F=\frac{2E}{1-v^{2}}\left(\frac{2}{3}c_{1}R^{1/2}h^{3/2}+\frac{1}{2}c_{2}h^{2}+\frac{1}{3}c_{3}R^{-1}h^{3}\right)$$

As already mentioned in the introduction, the constants $c_{1},\;c_{2},\;c_{3}$, for the domain $h_{max}/R\leq 1.32$, are $c_{1}=1.022$, $c_{2}=-0.1133$, $c_{3}=-0.0742$ [15]. The slope of the force—indentation curve at any given point is defined as the contact stiffness:

(10) $$\frac{dF}{dh}=\frac{2ER}{1-v^{2}}\left[c_{1}{\left(\frac{h}{R}\right)}^{1/2}+c_{2}\left(\frac{h}{R}\right)+c_{3}{\left(\frac{h}{R}\right)}^{2}\right]$$

The contact stiffness at the maximum indentation depth is presented below:

(11) $$S={\left.\frac{dF}{dh}\right|}_{h_{max}}\;S=\frac{2ER}{1-v^{2}}\left[c_{1}{\left(\frac{h_{max}}{R}\right)}^{1/2}+c_{2}\left(\frac{h_{max}}{R}\right)+c_{3}{\left(\frac{h_{max}}{R}\right)}^{2}\right]=\;\frac{2E}{1-v^{2}}\left(c_{1}R^{\frac{1}{2}}h_{max}^{\frac{1}{2}}+c_{2}h_{max}+c_{3}R^{-1}h_{max}^{2}\right)$$

Furthermore, the equation of the tangent line to the force–indentation curve at $h=h_{max}$ is given by:

(12) F=Sh+b

In Equation (12), b represents the point of intersection between the tangent line (12) and the force axis. By substituting $h=h_{max}$ into Equation (12), the value of b can be readily calculated:

(13) $$F_{max}=Sh_{max}+b=>\;b=F_{max}-Sh_{max}$$

In addition, using also Equations (9) and (11),

$$b=\frac{2E}{1-v^{2}}\left(\frac{2}{3}c_{1}R^{\frac{1}{2}}h_{max}^{\frac{3}{2}}+\frac{1}{2}c_{2}h_{max}^{2}+\frac{1}{3}c_{3}R^{-1}h_{max}^{3}\right)-\;\frac{2E}{1-v^{2}}\left(c_{1}R^{\frac{1}{2}}h_{max}^{\frac{3}{2}}+c_{2}h_{max}^{2}+c_{3}R^{-1}h_{max}^{3}\right)$$

Thus,

(14) $$b=-\frac{2E}{1-v^{2}}\left(\frac{1}{3}c_{1}R^{\frac{1}{2}}h_{max}^{\frac{3}{2}}+\frac{1}{2}c_{2}h_{max}^{2}+\frac{2}{3}c_{3}R^{-1}h_{max}^{3}\right)$$

Equation (14) leads to an interesting conclusion; the Young’s modulus can be easily calculated by plotting the tangent line of the force indentation curve at the point $h=h_{max}$. By utilizing a linear fit to the tangent line, b,S can be determined as fitting coefficients. Subsequently, the Young’s modulus can be easily calculated using Equation (14), assuming that the sample’s Poisson’s ratio is known.

The analysis can be extended by calculating the point of intersection between the tangent line (Equation (12)) and the indentation axis. At the point of intersection F=0, thus

(15) $$h_{com.}=-\frac{b}{S}$$

By combining Equations (11), (14) and (15), it is concluded

(16) $$h_{com.}=\frac{{\frac{1}{3}c}_{1}R^{\frac{1}{2}}h_{max}^{\frac{3}{2}}+\frac{1}{2}c_{2}h_{max}^{2}+{\frac{2}{3}c}_{3}R^{-1}h_{max}^{3}}{c_{1}R^{\frac{1}{2}}h_{max}^{\frac{1}{2}}+c_{2}h_{max}+c_{3}R^{-1}h_{max}^{2}}$$

Equation (16) yields an intriguing outcome. If the point of intersection between the tangent line and the indentation axis is determined, the indenter’s radius can be calculated using Equation (16). This outcome holds significance as the indenter’s radius can be calculated without requiring knowledge of the sample’s Young’s modulus. Therefore, by employing Equation (16), the indenter’s radius can be ascertained, followed by an easy calculation of the Young’s modulus using Equation (14). The procedure is also presented in Figure 1a and is summarized as follows. If $\frac{h_{max}}{R}\leq 1.32$, the force indentation data can be fitted to a simple equation of the form

(17) $$F=ah^{3/2}+bh^{2}+ch^{3},\;a>0,\;b<0,\;c<0$$

In Equation (17), a, b, c are fitting parameters. Subsequently, the tangent line (Equation (12)) is plotted at the point $h=h_{max}$ of the fitted curve. The factors b, S are determined as fitting parameters and the tip radius and the Young’s modulus are calculated using the Equations (16) and (14), respectively. Equations (14) and (16) represent simplified forms of the general case for $\frac{h_{max}}{R}\leq 1.32$ and are applicable to the majority of cases. In addition, it is straightforward to determine the suitability of the set of Equations (14) and (16), for the experiments by observing the nominal tip radius. For example, let us assume that the nominal tip radius provided by the manufacturer is 1 μm. If the maximum indentation depth is significantly smaller (e.g., ~0.5 μm), then it is safe to use the simplified Equations (14) and (16). However, it is significant to note that the method has no restrictions regarding the maximum indentation depth and it can be also applied for any $\frac{h_{max}}{R}$ ratio. Τhe general case is presented below:

(18) $$F=\frac{2E}{1-v^{2}}\left(\frac{2}{3}c_{1}R^{1/2}h^{3/2}+\frac{1}{2}c_{2}h^{2}+\frac{1}{3}c_{3}R^{-1}h^{3}+\cdots +\frac{1}{N}c_{N}R^{2-N}h^{N}\right)$$

For example, for $\frac{h_{max}}{R}\leq 4.9512$, N=6, $c_{1}=1.0100000,c_{2}=-0.0730300$, $c_{3}=-0.1357000$, $c_{4}=0.0359800$, $c_{5}=-0.0040240$, and $c_{6}=0.0001653$ [15] (as also mentioned in the introduction). Thus, in this case, the contact stiffness at $h=h_{max}$ is given by the following equation:

(19) $$S=\frac{2E}{1-v^{2}}\left(c_{1}R^{\frac{1}{2}}h_{max}^{\frac{1}{2}}+c_{2}h_{max}+c_{3}R^{-1}h_{max}^{2}+\cdots +c_{N}R^{2-N}h_{max}^{N-1}\right)$$

Furthermore, Equation (14) is also adjusted as shown below:

(20) $$b=-\frac{2E}{1-v^{2}}\left(\frac{1}{3}c_{1}R^{\frac{1}{2}}h_{max}^{\frac{3}{2}}+\frac{1}{2}c_{2}h_{max}^{2}+\frac{2}{3}c_{3}R^{-1}h_{max}^{3}+\cdots +\frac{N-1}{N}c_{N}R^{2-N}h_{max}^{N}\right)$$

Thus, the point of intersection between the tangent line and the indentation axis becomes

(21) $$h_{com.}=\frac{{\frac{1}{3}c}_{1}R^{\frac{1}{2}}h_{max}^{\frac{3}{2}}+\frac{1}{2}c_{2}h_{max}^{2}+{\frac{2}{3}c}_{3}R^{-1}h_{max}^{3}+\cdots +\frac{N-1}{N}c_{N}R^{2-N}h_{max}^{N}}{c_{1}R^{\frac{1}{2}}h_{max}^{\frac{1}{2}}+c_{2}h_{max}+c_{3}R^{-1}h_{max}^{2}+\cdots +c_{N}R^{2-N}h_{max}^{N-1}}$$

Furthermore, Equation (21) can be expressed as follows:

(22) $$\frac{h_{com.}}{R}=\frac{{\frac{1}{3}c}_{1}{\left(\frac{h_{max}}{R}\right)}^{3/2}+\frac{1}{2}c_{2}{\left(\frac{h_{max}}{R}\right)}^{2}+{\frac{2}{3}c}_{3}{\left(\frac{h_{max}}{R}\right)}^{3}+\cdots +\frac{N-1}{N}c_{N}{\left(\frac{h_{max}}{R}\right)}^{N}}{c_{1}{\left(\frac{h_{max}}{R}\right)}^{\frac{1}{2}}+c_{2}\left(\frac{h_{max}}{R}\right)+c_{3}{\left(\frac{h_{max}}{R}\right)}^{2}+\cdots +c_{N}{\left(\frac{h_{max}}{R}\right)}^{N-1}}$$

**Figure 2:**
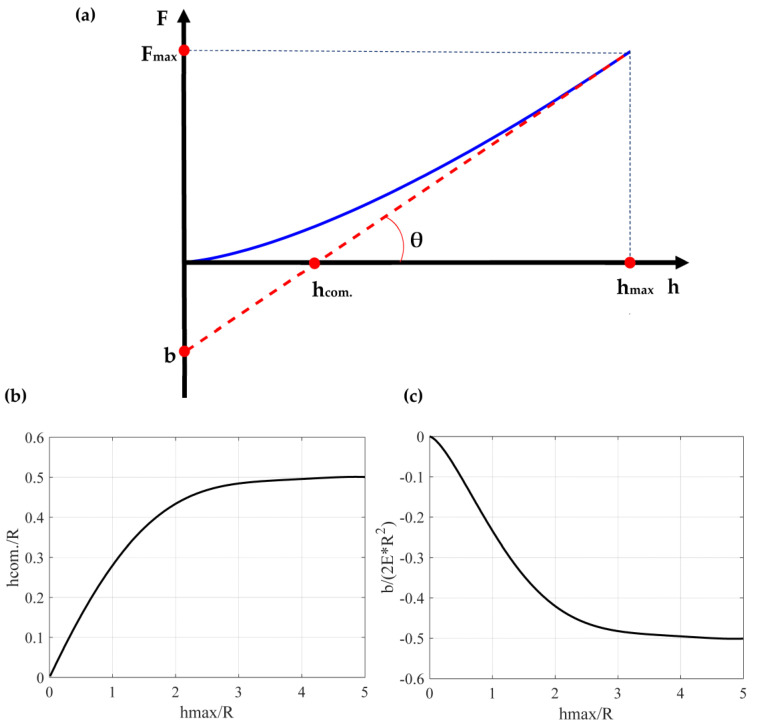
(**a**) The blue curve represents the force–indentation curve. The red dotted line represents the tangent line at the point $h=h_{max}$. The slope of the tangent line equals to the contact stiffness at $h=h_{max}$ (i.e., S=tan⁡(θ)). The slope, S, and the point of intersection between the tangent line and the force axis, denoted as b, are determined as fitting parameters. Subsequently, the indenter’s radius can be calculated using Equation (16). Lastly, the Young’s modulus is determined using Equation (14). (**b**) The $\frac{h_{com.}}{R}=f\left(\frac{h_{max}}{R}\right)$ function. (**c**) The $\frac{b}{2E^{*}R^{2}}=f\left(\frac{h_{max}}{R}\right)$ function.

The graphical representation of Equation (22) is presented in Figure 2b. It is interesting to note that for big $\frac{h_{max}}{R}$ ratios, $\frac{h_{com.}}{R}$ tends to a limit value which its equal to 0.5. In addition, Equation (20) can be also written in the form

(23) $$\frac{b}{2E^{*}R^{2}}=-\left[\frac{1}{3}c_{1}{\left(\frac{h_{max}}{R}\right)}^{3/2}+\frac{1}{2}c_{2}{\left(\frac{h_{max}}{R}\right)}^{2}+\frac{2}{3}c_{3}{\left(\frac{h_{max}}{R}\right)}^{3}+\cdots +\frac{N-1}{N}c_{N}{\left(\frac{h_{max}}{R}\right)}^{N}\right]$$

The $\frac{b}{2E^{*}R^{2}}=f\left(\frac{h_{max}}{R}\right)$ function is presented in Figure 2c. For big $\frac{h_{max}}{R}$ ratios the factor $\frac{b}{2E^{*}R^{2}}$ tends to −0.5. However, it is crucial to note that attaining the limit values is challenging in real experiments. For example, the probability of a plastic deformation is high in such cases. However, they present significant mathematical interest and can be used for a deeper understanding of the underlying theory. For example, assume that $\frac{h_{max}}{R}=5$. In this case, $\frac{h_{com.}}{R}=0.5$. This result can be used as a paradigm to realize that the relation between $h_{com.}$ and $h_{max}$ does not depend on the Young’s modulus value. This outcome is of paramount significance, as the method’s reliability is not contingent on the sample type when it comes to calculating R. It is also noteworthy that the independence of the relation between $h_{com.}$ and $h_{max}$ from the Young’s modulus applies to any indentation depth, as indicated by Equation (21).

### 2.2. AFM Indentation Experiments on an Agarose Gel

#### 2.2.1. Contact Point Determination

In AFM indentation experiments on biological samples, a critical aspect affecting result accuracy is the determination of the contact point between the tip and the sample. For precise determination of the contact point, the AtomicJ software (https://sourceforge.net/projects/jrobust/) was utilized [19]. The procedure is straightforward: each point of the curve is taken as a trial contact point, a polynomial is fitted to the precontact section, and the suitable contact model is applied to the force–indentation data [19]. The tested point that resulted in the lowest total sum of squares is considered as the contact point [19].

#### 2.2.2. Measurements

Spherical indenters (borosilicate glass spheres with Young’s modulus 64 GPa) were employed for the AFM indentation experiments (CP-PNPL-BSG-A, sQube, obtained from NanoAndMore GMBH, Wetzlar, Germany). The nominal tip radius of such an indenter, as specified by the manufacturer, is 1 μm with a deviation of ±10% (i.e., 0.9 μm≤R≤1.1 μm). The indenters were calibrated prior to the experiments using the AFM test grating TGT1 from NT-MDT Instruments. For precise quantitative measurements, it is necessary to calibrate the probe parameters. To calibrate the laser detection system’s sensitivity in terms of nanometer deflection per volt signal, a force vs. distance curve on mica was firstly obtained [20]. By positioning two cursors on the contact section of the force vs. distance curve, the deflection sensitivity was determined [20]. The spring’s constant determination was performed using the thermal noise method. The experiments were performed on agarose gels with concentration 2.5% in a 35 mm petri dish. The Poisson’s ratio of the agarose gel can be considered equal to v=0.5 due to the high-water content. The Young’s modulus was calculated through conventional fitting procedures using Equation (9) (and using also the measured R-value), as well as employing the method proposed in this paper (i.e., fitting the tangent line to Equation (12) and subsequently employing Equation (16) to determine the tip radius and (14) to determine the Young’s modulus).

## 3. Results

**Figure 3:**
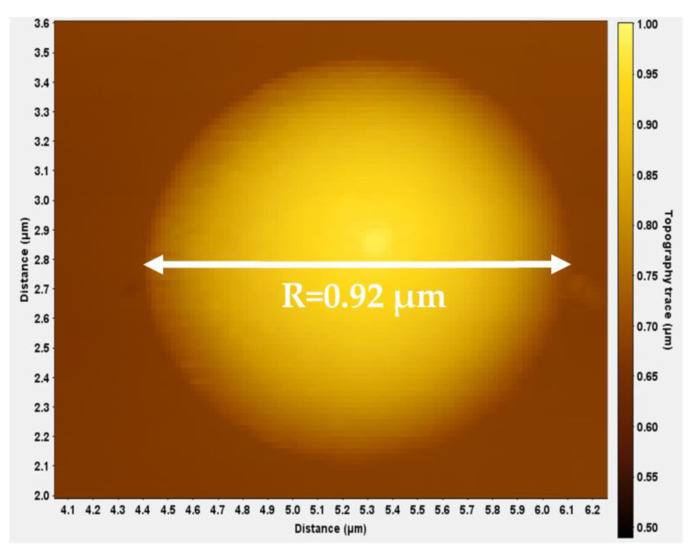
Calibration of the spherical tip using the AFM grating. The tip radius resulted in R=0.92 μm.

**Figure 4:**
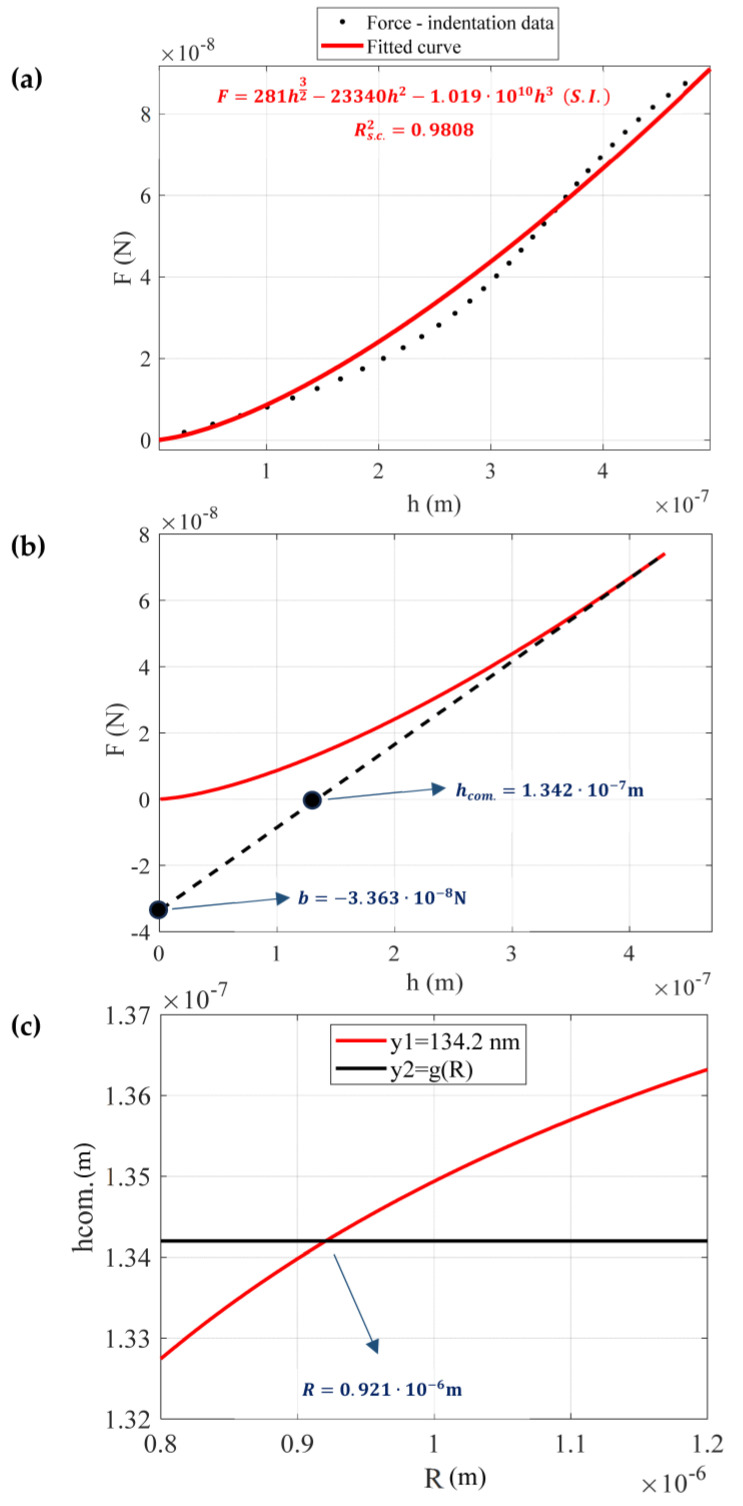
A paradigm of the proposed method. (**a**) The force-indentation data were fitted to Equation (17) ($F=281h^{3/2}+23340h^{2}+1.019\cdot 10^{10}h^{3},\;R_{s.c.}^{2}=0.9808$). (**b**) The tangent line at the maximum indentation depth $h=h_{max}=432\;nm$ was plotted. The point of intersection between the line and the F-axis is $b=-3.363\cdot 10^{-8}\;N$ and the point of intersection between the line and the h-axis is $h_{com.}=1.342\cdot 10^{-7}\;m$. (**c**) A graphical solution of Equation (26).

An example illustrating the application of the proposed method is provided below. The experiment was performed using a spherical tip with a nominal radius equal to 1 μm. The tip was calibrated using an AFM grating as described in Section 2.2.2, and the result was $R_{meas.}=0.92\;\mu m$ (this result is within the ±10% range provided by the manufacturer, $0.9\;\mu m\leq R_{meas.}\leq 1.1\;\mu m$). The calibration of the spherical indenter using the AFM grating is presented in Figure 3. The force-indentation data are shown in Figure 4a. The data were fitted to Equation (17):

(24) $$F=281h^{3/2}-23340h^{2}-1.019\cdot 10^{10}h^{3}\;(S.I.)$$

The R-squared coefficient resulted in $R_{s.c.}^{2}=0.9808$. The force-indentation data and the fitted curve (Equation (24)) are presented comparatively in Figure 4a. Subsequently, the tangent line at $h=h_{max}=432\;nm$ was plotted.

The tangent line is described by the following Equation (Figure 4b):

(25) $$F_{tang.}=0.2505h-3.363\cdot 10^{-8}\;(S.I.)$$

Thus, S=0.2505 N/m, $b=-3.363\cdot 10^{-8}\;N$ and $h_{com.}=-\frac{b}{S}=1.342\cdot 10^{-7}\;m$. In addition, Equation (16) is written as follows:

(26) $$1.342\cdot 10^{-7}=\frac{\frac{1}{3}1.022{\left(43.2\cdot 10^{-8}\right)}^{\frac{3}{2}}R^{\frac{1}{2}}-\frac{1}{2}0.1133{\left(43.2\cdot 10^{-8}\right)}^{2}-\frac{2}{3}0.0742{\left(43.2\cdot 10^{-8}\right)}^{3}R^{-1}}{1.022{\left(43.2\cdot 10^{-8}\right)}^{\frac{1}{2}}R^{\frac{1}{2}}-0.1133\cdot 43.2\cdot 10^{-8}-0.0742{\left(43.2\cdot 10^{-8}\right)}^{2}R^{-1}}$$

Equation (26) can be easily solved using any basic software (e.g., Matlab). The tip radius resulted in $R=0.921\cdot 10^{-6}\;m=0.921\;\mu m$. This result is nearly identical to the value that was measured using the AFM grating.

A graphical solution of Equation (26) is also presented in Figure 4c. In particular, the functions

$y_{1}(R)=1.342\cdot 10^{-7}m$ and

$$y_{2}\left(R\right)=\frac{\frac{1}{3}1.022{\left(43.2\cdot 10^{-8}\right)}^{\frac{3}{2}\;}R^{\frac{1}{2}}-\frac{1}{2}0.1133{\left(43.2\cdot 10^{-8}\right)}^{2}-\frac{2}{3}0.0742{\left(43.2\cdot 10^{-8}\right)}^{3}R^{-1}}{1.022{\left(43.2\cdot 10^{-8}\right)}^{\frac{1}{2}}R^{\frac{1}{2}}-0.1133\cdot 43.2\cdot 10^{-8}-0.0742{\left(43.2\cdot 10^{-8}\right)}^{2}R^{-1}}$$

were plotted within the domain $0.8\cdot 10^{-6}\;m\leq R\leq 1.2\cdot 10^{-6}\;m$. The common point of the two functions is $R=0.921\cdot 10^{-6}\;m$. In addition, it is also easy to calculate the Young’s modulus using Equation (14):

(27) $$3.363\cdot 10^{-8}=\frac{2E}{1-0.5^{2}}\left[\frac{1}{3}1.022{\left(43.2\cdot 10^{-8}\right)}^{\frac{3}{2}}{\left(0.921\cdot 10^{-6}\right)}^{\frac{1}{2}}-\frac{1}{2}0.1133{\left(43.2\cdot 10^{-8}\right)}^{2}\;-\frac{2}{3}0.0742{\left(43.2\cdot 10^{-8}\right)}^{3}{\left(0.921\cdot 10^{-6}\right)}^{-1}\right]$$

**Figure 5:**
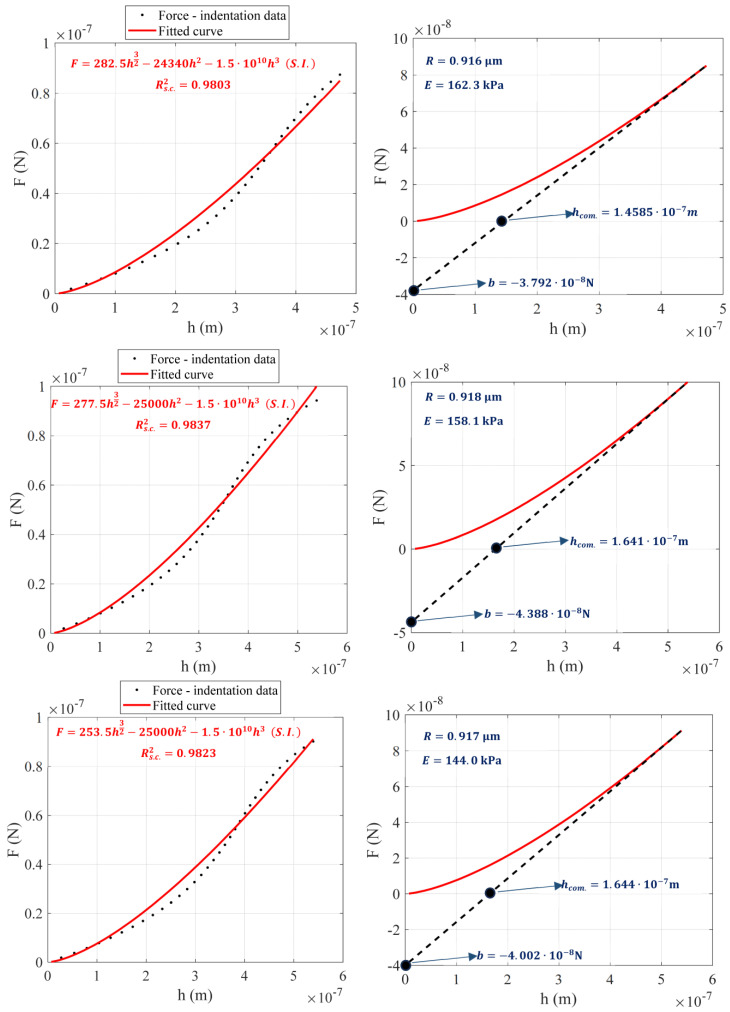
Three additional paradigms of the proposed method.

**Figure 6:**
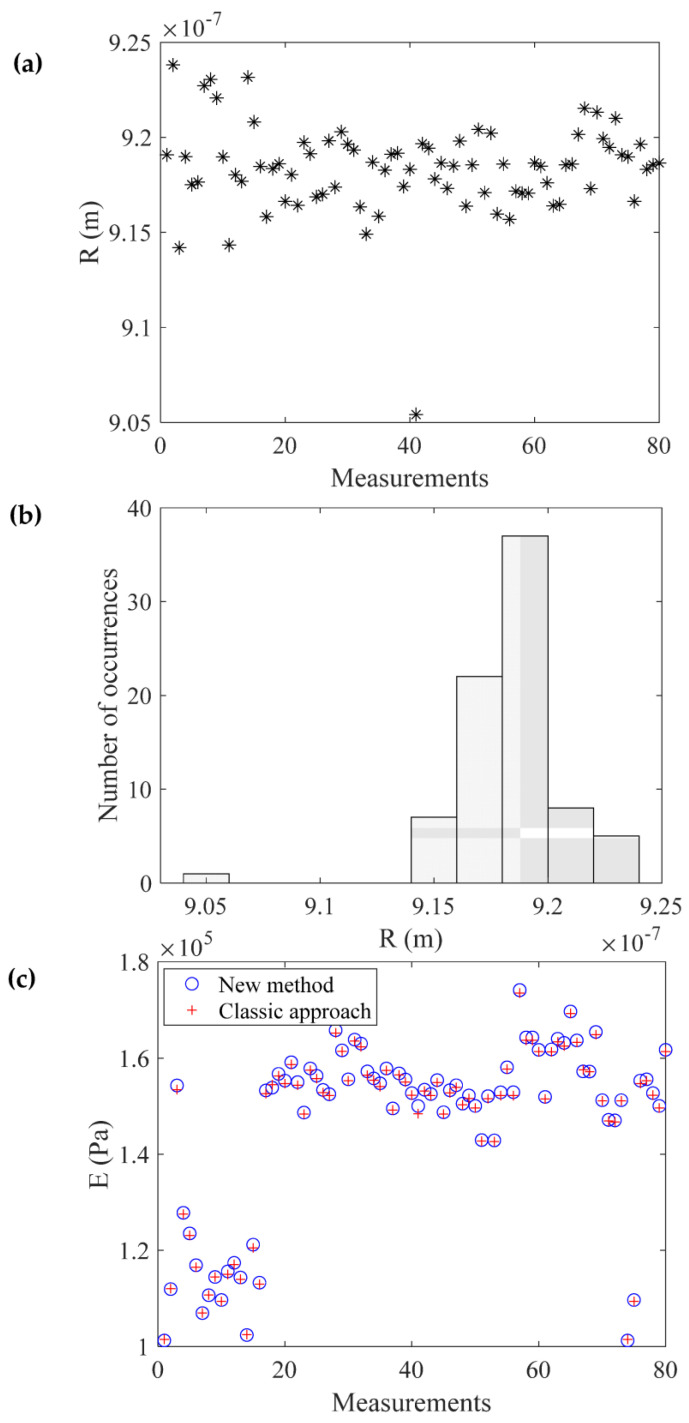
Evaluating the reliability of the proposed method. (**a**) The determination of the indenter’s radius using 80 measurements. The mean ± standard deviation value obtained was 0.9184 μm ± 0.0135 μm, which agrees with the tip radius measurement obtained using the AFM grating. (**b**) A histogram constructed using the values presented in (**a**). (**c**) The Young’s modulus was calculated using the method proposed in this paper and a conventional fitting procedure. The results from the two methods were nearly identical.

By solving Equation (27), the Young’s modulus resulted in E=132.5 kPa. Three additional paradigms are also presented in Figure 5. To validate the accuracy of the method, an additional 76 force–indentation curves were also processed. The results are shown in Figure 6. Figure 6a displays the outcomes concerning the tip radius. The mean ± standard deviation value resulted in 0.9184 μm±0.0135 μm, which is in agreement with the result obtained using the AFM grating. A histogram of the R-values is also presented in Figure 6b. In Figure 6c, the Young’s modulus values as calculated using the proposed by this paper approach (Equations (14) and (16)) and using a classic fitting procedure are presented for comparison. The results are nearly identical.

## 4. Discussion

**Figure 7:**
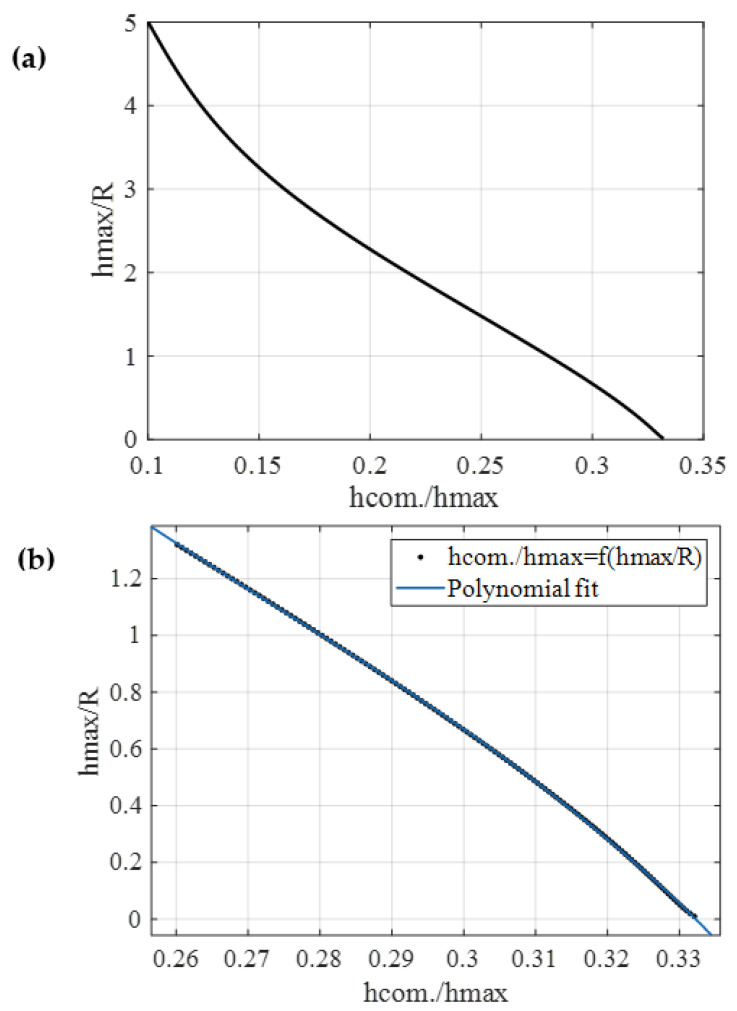
(**a**) The data for $\frac{h_{max}}{R}=f\left(\frac{h_{com.}}{h_{max}}\right)$ in the domain 0.05≤h/R≤5.00. (**b**) The same data within the domain 0≤h/R≤1.32. The data were fitted to a fourth-degree polynomial function (Equation (28)).

In this paper, a new method is presented that allows for the determination of the Young’s modulus of soft biological materials without the need for an experimental calibration procedure for the AFM tip. When using spherical indenters, the radius of the indenter can be readily calculated by employing the tangent line of the fitted curve of the force–indentation data at $h=h_{max}$. More specifically, the point of intersection between the tangent line and the indentation axis can unveil the value of R (as defined in Equations (16) and (21)). Subsequently, by utilizing the point of intersection of the tangent line with the force axis, the Young’s modulus can be readily determined (as described in Equations (14) and (20)). The proposed approach is reliable, as the results were nearly identical to those obtained using conventional methods, such as tip calibration through an AFM grating and Young’s modulus determination using traditional fitting procedures. The application of the new method requires solving Equations (16) and (14) (or (21) and (20) in the general case). Furthermore, utilizing Equation (22), an equation that establishes a numerical relationship between the ratio $\frac{h_{com.}}{h_{max}}$ and the ratio $\frac{h_{max}}{R}$ can be derived. The $\frac{h_{max}}{R}=f\left(\frac{h_{com.}}{h_{max}}\right)$ data are presented in Figure 7a. This is a noteworthy finding, as the data can be fitted to a polynomial curve to derive a straightforward equation that establishes a relationship between $\frac{h_{com.}}{h_{max}}$ with $\frac{h_{max}}{R}$ within the specified domain of interest.

For example, the data $\frac{h_{max}}{R}=f\left(\frac{h_{com.}}{h_{max}}\right)$ were fitted to the function (for the domain 0≤h/R≤1.32):

(28) $$\frac{h_{max}}{R}=p_{4}{\left(\frac{h_{com.}}{h_{max}}\right)}^{4}+p_{3}{\left(\frac{h_{com.}}{h_{max}}\right)}^{3}+p_{2}{\left(\frac{h_{com.}}{h_{max}}\right)}^{2}+p_{1}\frac{h_{com.}}{h_{max}}+p_{0}$$

The fit was perfect ($R_{s.c.}^{2}=1.0000$). The fitting coefficients are as follows: $p_{4}=-8149,\;p_{3}=8816$, $p_{2}=-3592$ and $p_{1}=637.3$ and $p_{0}=-39.27.$ This is a significant result, as Equation (28) can be used to determine the tip radius after graphically estimating the point $h_{com.}$ For example, let us consider the case introduced at the beginning of the results section (depicted in Figure 4). In this example, $h_{com.}=134.2\;nm$ and $h_{max}=432\;nm$. Thus, $\frac{h_{com.}}{h_{max}}=0.3106$. By employing Equation (28), it is straightforward to calculate that $\frac{h_{max}}{R}=0.4691$. Thus, R=0.921 μm. Furthermore, to enhance the ease of applying the proposed method, two tables presenting the data for $\frac{h_{max}}{R}=f\left(\frac{h_{com.}}{h_{max}}\right)$ are also provided. In Table 1, the values of $\frac{h_{max}}{R}=f\left(\frac{h_{com.}}{h_{max}}\right)$ are presented within the domain of 0.01≤h/R≤1.32, and in Table 2, within the domain 0.05≤h/R≤5.00.

It is also noteworthy to emphasize that a ‘rational approach’ would involve concurrently determining the tip radius and the sample’s Young’s modulus by employing a simple fit to Equation (9), under the assumption of $h_{max}/R\leq 1.32$. The reason is that Equation (9) can be written as follows:

(29) $$F=\frac{4c_{1}ER^{1/2}}{3(1-v^{2})}h^{3/2}+\frac{c_{2}E}{\left(1-v^{2}\right)}h^{2}+\frac{2c_{3}ER^{-1}}{3(1-v^{2})}h^{3}$$

By combining Equations (17) and (29), it is concluded

(30) $$a=\frac{4c_{1}ER^{1/2}}{3(1-v^{2})}$$

(31) $$b=\frac{c_{2}E}{\left(1-v^{2}\right)}$$

(32) $$c=\frac{2c_{3}ER^{-1}}{3(1-v^{2})}$$

Thus, given that the coefficients $c_{1},c_{2},c_{3}$ are known, it may be assumed that the Young’s modulus can be calculated using Equation (31), and subsequently, employing Equation (30) or Equation (32), the tip radius can also be determined. However, this is not accurate; there exist various combinations of the fitting coefficients a, b, and c that can result in the same curve. This fact can be also proved using Equation (24). In this case, $b=23,340\;\frac{N}{m^{2}}.$ Therefore, utilizing Equation (31), $E=2.398\cdot 10^{5}\;Pa.$ In addition, $a=281\;\frac{N}{m^{3/2}}$. Therefore, by applying Equation (30), $R=0.225\cdot 10^{-6}\;m$, which is approximately four times smaller than the actual value (0.921 μm). Hence, it is imperative to adhere to the procedure outlined in this paper for the calculation of R and E.

The precise equations concerning deep spherical indentations are typically circumvented in experiments involving soft biological materials, as fitting the data to Equation (1) is simpler and the errors in Young’s modulus calculations for $h_{max}/R<1$ are not substantial. However, this paper demonstrates that Equations (7) and (8) can provide significantly more options compared to Equation (1). Indeed, these equations can lead to the determination of the indenter’s radius using a simple force–indentation curve. If we use Equation (1) instead of Equation (7), the contact stiffness becomes

(33) $$S=\frac{2E}{1-v^{2}}R^{\frac{1}{2}}h_{max}^{\frac{1}{2}}$$

In this case,

(34) $$b=F_{max}-Sh_{max}=\frac{4E}{3\left(1-v^{2}\right)}R^{\frac{1}{2}}h_{max}^{\frac{3}{2}}-\frac{2E}{1-v^{2}}R^{\frac{1}{2}}h_{max}^{\frac{3}{2}}=\frac{-2E}{3\left(1-v^{2}\right)}R^{\frac{1}{2}}h_{max}^{\frac{3}{2}}$$

Subsequently, the point of intersection between the tangent line and the indentation axis can be calculated:

(35) $$h_{com.}=-\frac{b}{S}=\frac{h_{max}}{3}$$

Hence, when utilizing Equation (1), it becomes impossible to calculate R, as the values for $h_{com.}$ consistently equate to $\frac{h_{max}}{3}$. Therefore, in cases for which $h_{max}\ll R$ and Equation (1) accurately describe the data, the method cannot be applied.

It is also crucial to highlight the significant reliability of the proposed method. The experiments were conducted on an agarose gel at arbitrarily selected points. The calculated Young’s moduli were within the range of 102 kPa ≤E ≤174 kPa (see also Figure 6c). However, despite the significant variation in the Young’s modulus, there is only a slight variation in the calculation of the indenter’s radius (as clearly depicted in Figure 6a). If we were to test a hypothetically perfect elastic half-space, the force–indentation data and the fitted curve would be identical, and the calculation of R would be consistent across all force–indentation curves. However, for real soft samples, the data do not perfectly follow Equation (7). Consequently, errors in $h_{com.}$ and b would emerge, leading to variations in R calculations. Nevertheless, the main outcome is that even though there exists a disparity between the fitted curve and the data in all instances, the inaccuracies pertaining to the tip radius calculation were exceedingly minor. Specifically, the computed values in each measurement, as depicted in Figure 6a, closely resembled the measured values obtained through the AFM grating (as shown in Figure 3). This is a logical outcome when we consider Equation (22). This equation suggests that there is a specific relationship between $h_{com.}$ and $h_{max}$ that can lead to the calculation of the ratio $h_{max}/R$ and, as a result, to the calculation of the indenter’s radius (see also Table 1 and Table 2). Importantly, this relationship remains uninfluenced by the Young’s modulus. When collecting force–indentation data, $h_{max}$ is a known parameter and determining $h_{com.}$ becomes straightforward by utilizing the tangent line to the force–indentation curve at the maximum indentation depth. Thus, the ratio $h_{com.}/h_{max}$ unveils the ratio $\frac{h_{max}}{R},$ consequently determining the radius of the indenter. Furthermore, it is worth noting that by multiplying $h_{com.}/h_{max}$ with $h_{max}/R$ in Table 1 and Table 2, a new table can be generated that establishes a relationship between $h_{com.}$ and R.

In addition, it is also important to note that most of the cells and biological tissues present a viscoelastic behavior [21,22]. However, for small indentation rates, the elastic models derived from Hertzian mechanics can be employed for data fitting [22]. From this perspective, our method remains valid for viscoelastic materials. By utilizing an extremely small indentation rate, we can determine the indenter’s radius (R). Subsequently, we can employ the R-value for dynamic loading at higher indentation rates, allowing us to extract the viscoelastic properties of the material. However, a very interesting question is whether it is possible to calculate R using force–indentation curves for different loading conditions [23,24]. This constitutes a fascinating task for future research.

It is also noteworthy to emphasize that the development of algorithms for intelligent micro- and nano-systems is crucial for technological progress and its applications in medicine and biology. AFM processes represent cutting-edge research in today’s context, offering numerous possibilities for potential clinical applications in the future. However, in order to attain this objective, it is crucial to develop straightforward and automated procedures for data processing. Simplifying experimental procedures through algorithms based on mathematical criteria is crucial for the utilization of AFM technology in medical applications, such as disease diagnosis.

## 5. Conclusions

This paper introduces a novel method for calibrating the indenter in AFM nanoindentation experiments involving soft materials using spherical indenters. The calibration of the indenter is founded on processing the force–indentation curve employing rigorous mathematical criteria. Hence, it becomes possible to calculate the Young’s modulus and the AFM tip radius using the force–indentation data without requiring any additional experimental procedures. The fundamental steps of the method are outlined as follows:

- Fit the force–indentation data to Equation (17).
- Plot the tangent line of the fitted curve at the maximum indentation depth.
- Determine the point of intersection between the tangent line and the indentation axis and solve either (16) or (21). Alternatively, for simplification, employ Equation (28) or refer to Table 1 and Table 2.
- To calculate the Young’s modulus at the tested point, identify the intersection point between the tangent line and the force axis, and solve either Equations (14) or (20).

The proposed approach can be integrated into typical AFM equipment to automate and streamline the experimental procedures. The development of intelligent AFM systems increases the potential for utilizing AFM processes in practical clinical applications, such as disease diagnosis.

## Figures and Tables

**Table 1:** The $h_{com.}/h_{max}$ ratio for the domain $0.01\leq h_{max}/R\leq 1.00$.

$h_{max}/R$	$h_{com.}/h_{max}$	$h_{max}/R$	$h_{com.}/h_{max}$	$h_{max}/R$	$h_{com.}/h_{max}$
0.01	0.3321	0.35	0.3168	0.68	0.2993
0.02	0.3315	0.36	0.3163	0.69	0.2987
0.03	0.3310	0.37	0.3159	0.70	0.2982
0.04	0.3305	0.38	0.3154	0.71	0.2976
0.05	0.3301	0.39	0.3149	0.72	0.2970
0.06	0.3297	0.40	0.3144	0.73	0.2964
0.07	0.3292	0.41	0.3139	0.74	0.2959
0.08	0.3288	0.42	0.3134	0.75	0.2953
0.09	0.3284	0.43	0.3128	0.76	0.2947
0.10	0.3280	0.44	0.3123	0.77	0.2941
0.11	0.3276	0.45	0.3118	0.78	0.2935
0.12	0.3272	0.46	0.3113	0.79	0.2929
0.13	0.3268	0.47	0.3108	0.80	0.2924
0.14	0.3263	0.48	0.3103	0.81	0.2918
0.15	0.3259	0.49	0.3097	0.82	0.2912
0.16	0.3255	0.50	0.3092	0.83	0.2906
0.17	0.3251	0.51	0.3087	0.84	0.2900
0.18	0.3246	0.52	0.3081	0.85	0.2894
0.19	0.3242	0.53	0.3076	0.86	0.2888
0.20	0.3238	0.54	0.3071	0.87	0.2882
0.21	0.3233	0.55	0.3065	0.88	0.2876
0.22	0.3229	0.56	0.3060	0.80	0.2870
0.23	0.3225	0.57	0.3054	0.90	0.2864
0.24	0.3220	0.58	0.3049	0.91	0.2858
0.25	0.3215	0.59	0.3043	0.92	0.2852
0.26	0.3211	0.60	0.3038	0.93	0.2846
0.27	0.3206	0.61	0.3032	0.94	0.2840
0.28	0.3202	0.62	0.3027	0.95	0.2834
0.29	0.3197	0.63	0.3021	0.96	0.2827
0.30	0.3192	0.64	0.3016	0.97	0.2821
0.31	0.3188	0.65	0.3010	0.98	0.2815
0.32	0.3183	0.66	0.3004	0.99	0.2809
0.33	0.3178	0.67	0.2999	1.00	0.2802
0.34	0.3173				

**Table 2:** The $h_{com.}/h_{max}$ ratio for the domain $0.05\leq h_{max}/R\leq 5.00$.

$h_{max}/R$	$h_{com.}/h_{max}$	$h_{max}/R$	$h_{com.}/h_{max}$	$h_{max}/R$	$h_{com.}/h_{max}$
0.05	0.3301	1.75	0.2326	3.40	0.1443
0.10	0.3280	1.80	0.2294	3.45	0.1424
0.15	0.3259	1.85	0.2263	3.50	0.1405
0.20	0.3238	1.90	0.2231	3.55	0.1386
0.25	0.3216	1.95	0.2200	3.60	0.1368
0.30	0.3192	2.00	0.2169	3.65	0.1351
0.35	0.3168	2.05	0.2138	3.70	0.1334
0.40	0.3144	2.10	0.2107	3.75	0.1317
0.45	0.3118	2.15	0.2077	3.80	0.1301
0.50	0.3092	2.20	0.2047	3.85	0.1285
0.55	0.3065	2.25	0.2017	3.90	0.1269
0.60	0.3039	2.30	0.1988	3.95	0.1254
0.65	0.3010	2.35	0.1959	4.00	0.1240
0.70	0.2982	2.40	0.1930	4.05	0.1225
0.75	0.2953	2.45	0.1901	4.10	0.1211
0.80	0.2924	2.50	0.1873	4.15	0.1198
0.85	0.2894	2.55	0.1845	4.20	0.1185
0.90	0.2864	2.60	0.1818	4.25	0.1172
0.95	0.2834	2.65	0.1791	4.30	0.1159
1.00	0.2803	2.70	0.1765	4.35	0.1147
1.05	0.2772	2.75	0.1739	4.40	0.1134
1.10	0.2741	2.80	0.1713	4.45	0.1122
1.15	0.2709	2.85	0.1688	4.50	0.1111
1.20	0.2678	2.90	0.1663	4.55	0.1099
1.25	0.2646	2.95	0.1639	4.60	0.1088
1.30	0.2614	3.00	0.1616	4.65	0.1077
1.35	0.2582	3.05	0.1592	4.70	0.1066
1.40	0.2550	3.10	0.1570	4.75	0.1055
1.45	0.2518	3.15	0.1547	4.80	0.1044
1.50	0.2486	3.20	0.1525	4.85	0.1033
1.55	0.2454	3.25	0.1504	4.90	0.1023
1.60	0.2422	3.30	0.1483	4.95	0.1012
1.65	0.2390	3.35	0.1463	5.00	0.1002
1.70	0.2358				

## Data Availability

Not applicable.
